# Patterns of Sexualized Drug Use among Gay, Bisexual, and Other Men Who Have Sex with Men Living with HIV: Insights from a Comprehensive Study—The U-SEX-2 GESIDA 9416 Study

**DOI:** 10.3390/jcm12237293

**Published:** 2023-11-24

**Authors:** Pablo Ryan, Helen Dolengevich-Segal, Luis Ramos-Ruperto, Alfonso Cabello, Matilde Sanchez-Conde, Jorge Vergas, Jorge Valencia, Guillermo Cuevas, José Sanz, Javier Curto-Ramos, Javier Pérez-Bootello, Jorge Naharro-Rodriguez, Mar J. F. Ollero, Lucio Garcia Fraile, Leire Pérez-Latorre, Otilia Bisbal, Sara De la Fuente, Juan Emilio Losa, Miguel Cervero, Miriam Estebanez, Inés Suarez-Garcia, Alejandra Gimeno, Ignacio Terrancle, Rafael Mican, Alicia González-Baeza

**Affiliations:** 1 Internal Medicine and HIV Unit, Infanta Leonor University Hospital, 28031 Madrid, Spain; 2 Department of Medicine, Medicine Faculty, Complutense University of Madrid (UCM), 28040 Madrid, Spain; 3 Biomedical Research Network Center in Infectious Diseases (CIBERINFEC), Instituto de Salud Carlos III, 28034 Madrid, Spain; 4 Dual Pathology Program, Henares Hospital, 28822 Coslada, Spain; 5 Department of Psychiatry, Francisco de Vitoria University, 28822 Madrid, Spain; 6 Infectious Diseases and HIV Unit, La Paz University Hospital, IdiPAZ, 28046 Madrid, Spain; 7 Infectious Diseases and HIV Unit, Hospital Universitario Fundación Jimenez Diaz y IIS-FJD, 28040 Madrid, Spain; 8 Department of Biological and Health Psychology, Autonomous University of Madrid (UAM), 28049 Madrid, Spainalicia.gonzalezb@uam.es (A.G.-B.); 9 Infectious Diseases and HIV Unit, Ramon y Cajal University Hospital, 28034 Madrid, Spain; 10 Internal Medicine Department, San Carlos University Hospital, 28040 Madrid, Spain; 11 Harm Reduction Unit “SMASD”, 28020 Madrid, Spain; 12 Internal Medicine, Alcalá University Hospital, 28805 Madrid, Spain; 13 Department of Psychiatry, Clinical Psychology and Mental Health, La Paz University Hospital, 28046 Madrid, Spain; 14 Dermatology Service, Ramon y Cajal University Hospital, 28034 Madrid, Spain; 15 Internal Medicine and HIV Unit, La Princesa University Hospital, 28006 Madrid, Spain; 16 HIV Unit, Gregorio Marañon University Hospital and IiSGM, 28009 Madrid, Spain; 17 Internal Medicine and HIV Unit, 12 de Octubre University Hospital, 28041 Madrid, Spain; 18 Internal Medicine and HIV Unit, Puerta del Hierro University Hospital, 28222 Majadahonda, Spain; 19 Infectious Diseases, Fundación Alcorcón University Hospital, 28922 Alcorcón, Spain; 20 Internal Medicine, Severo Ochoa University Hospital, 28914 Leganes, Spain; 21 Infectious Diseases and HIV Unit, Gómez Ulla University Hospital, 28047 Madrid, Spain; 22 Internal Medicine, Infanta Sofia University Hospital, Fundación para la Investigación e Innovación Biomédica del Hospital Universitario Infanta Sofía y Hospital Universitario del Henares (FIIB HUIS HHEN), 28702 San Sebastian de los Reyes, Spain; 23 Department of Medicine, Medicine Faculty, European University, 28032 Madrid, Spain; 24 Internal Medicine, Torrejón University Hospital, 28850 Torrejón de Ardoz, Spain; 25 Internal Medicine, Tajo University Hospital, 28670 Aranjuez, Spain

**Keywords:** MSM, high-risk sexual behaviors, HIV, sexualized drug use, chemsex, sexually transmitted infections

## Abstract

Background: Sexualized drug use (SDU) has become a public health concern in recent years. This study aimed to estimate the prevalence of SDU in gay, bisexual, and other men who have sex with men living with HIV (HIV + GBMSM) in Madrid during 2019/2020 and compare it with data from 2016/2017 in order to detect changes in patterns. Methods: We analyzed the frequency of SDU in a sample of HIV + GBMSM attending HIV clinics, who participated in an anonymous online survey regarding sexual behavior and recreational drug use. The association between SDU, sexual risk behaviors, and STIs was evaluated. Results: This study included 424 HIV + GBMSM, with a mean age of 40 (10.43) years. Overall, 94% (396) reported being sexually active. Additionally, 33% (140) had been diagnosed with an STI within the previous year. Moreover, 54% (229) had used drugs in the last year, 25% (107) engaged in SDU, and 16% (17) reported engagement in slamsex. After adjusting for confounding factors, SDU was associated with STIs, fisting, unprotected anal intercourse, and having >24 sexual partners in the last year. According to the DUDIT test scores, 80% (81) probably had problematic drug use (≥6 points), and 8% (8) probable drug dependence (≥25 points). When comparing the U-SEX-1 (2016/2017) data with the U-SEX-2 (2019/2020) data, no significant differences were found in the proportion of participants practicing SDU or slamming. Conclusions: The prevalence of SDU among HIV + GBMSM has remained high in recent years and without significant changes. The risk of problematic drug use among those who practice SDU is high. We observed a clear association between SDU, high-risk sexual behaviors, and STIs.

## 1. Introduction

Sexualized drug use (SDU), also known as *chemsex,* refers to the use of drugs to enhance sexual experiences [1]. Commonly used drugs in SDU include mephedrone and crystal methamphetamine, known for their arousal and stamina-enhancing effects, as well as γ-hydroxybutyrate (GHB)/γ-butyrolactone (GBL) and ketamine, which have disinhibiting properties. Injecting drugs in the context of SDU is referred to as slamsex [2].

SDU is relatively prevalent among gay, bisexual, and other men who have sex with men (GBMSM) attending sexual health clinics [3] and appears to be more common among people living with human immunodeficiency virus (HIV) [4]. SDU has been linked to high-risk sexual practices such as unprotected anal intercourse (UAI) and engaging in sexual activities with multiple partners, which can lead to sexually transmitted infections (STIs), including those caused by blood-borne viruses such as HIV and hepatitis C [4].

SDU is a phenomenon strongly influenced by social networks and it has been observed in major cities worldwide for several years. In Spain, it is particularly prominent in Madrid and Barcelona [5]. The U-SEX-1 study conducted in Madrid in 2016 reported a prevalence of SDU among gay, bisexual, and other men who have sex with men living with HIV (HIV + GBMSM) of 29% [6].

Some studies from various geographic regions have investigated the extent of SDU among HIV + GBMSM [4,6]. These studies share a similar methodology, collecting data through online anonymous self-completed surveys. Research has demonstrated that SDU has detrimental effects on the physical and psychological health of GBMSM, and due to its association with risky sexual behaviors and STIs, it poses significant public health concerns [7].

In response to these findings, institutions, and non-governmental organizations (NGOs) have implemented strategies for early detection, risk reduction, and harm reduction in HIV+ GBMSM. Awareness campaigns have been launched by NGOs, STI centers, and HIV clinics. Additionally, strategies for identifying problematic drug use and creating alternative support systems for individuals affected by SDU or slamsex have been established [8]. As SDU is a relatively new phenomenon, its evolution remains ongoing, necessitating vigilance to identify emerging behavioral patterns that may increase the risk of HIV transmission, other STIs, and severe mental health issues.

The present study aims to investigate the prevalence of SDU, patterns of drug consumption, sexual practices, and psychological distress symptoms in a sample of HIV + GBMSM. Furthermore, we compare our findings with the data collected four years prior in the USEX-1 study to examine changes over time.

## 2. Materials and Methods

The U-SEX-2 GESIDA 9416 study was conducted in 20 hospitals within the Madrid area over a planned duration of 6 months. Participants who met the inclusion criteria (GBMSM aged ≥18 years with documented HIV infection) were invited to participate in the study by Infectious Diseases Specialists at the HIV clinics of each participating hospital. The methodology employed in the U-SEX-2 study (2019/2020) was consistent with that of the U-SEX-1 study (2016/2017), which was conducted four years earlier and published in two articles [6,9]. The U-SEX-2 GESIDA 9416 study commenced in October 2019 and was prematurely halted due to the coronavirus disease (COVID-19) pandemic in February 2020.

The purpose of the study was explained to the patients by physicians, who then extended an invitation to participate. Patients were provided with a unique code and a link to an online survey via a non-transferable card. Information about the study was printed on the back of the card. The survey was self-completed by the participants. To assess response rates and sample representativeness, physicians entered a series of data into a local database, including the participant’s code, age, level of education, nationality, and previous or current drug use (Table 1). This study adheres to the Strengthening the Reporting of Observational Studies in Epidemiology (STROBE) guidelines.

The online survey evaluated various domains, including general sociodemographic data, HIV infection status, sexual risk practices, STI diagnosis, psychiatric disorders, and history of drug use. If participants reported any form of drug use, they were directed to a second part of the survey, which evaluated the types of drugs used, the context in which they were used, frequency, route of administration, and other SDU-related variables. Inquiries were made regarding the use of apps for seeking sexual contacts or for searching for drugs. The U-SEX-2 study (2019/2020) utilized the same variables and definitions as the U-SEX-1 study (2016/2017) [6].

Definitions and scales

SDU was defined as the intentional use of mephedrone or similar cathinones, 3,4-methylenedioxy-N-methylamphetamine (MDMA), methamphetamine, amphetamines, GHB/GBL, ketamine, or cocaine during sexual activity within the previous year. The questionnaires inquired about the use of the following drugs: synthetic cathinones like mephedrone, methamphetamine, ketamine, cocaine, GHB/GBL, flunitrazepam, heroin, amphetamines, MDMA/ecstasy, hallucinogens (LSD, mushrooms…), speed, cannabis, synthetic cannabinoids, poppers, tobacco, and alcohol. Information regarding adherence to antiretroviral therapy (ART) was self-reported by participants. It was recorded as the number of days the medication was not taken in the last month. Incomplete adherence refers to having more than three missed doses per month. Psychological distress was measured using the Hospital Anxiety and Depression Scale (HADS), a 14-item scale with two 7-item subscales assessing anxious and depressive symptoms. A score above 8 on the subscales indicated significant symptoms of depression or anxiety [10,11]. The Drug Use Disorders Identification Test (DUDIT), an 11-item self-administered screening instrument, was also employed. It provided information on the level of drug intake and selected criteria for substance abuse/harmful use and dependence based on the ICD-10 and DSM-4 diagnostic systems [12]. DUDIT scores ≥6 suggested probable drug-related problems, while scores ≥25 indicated probable drug dependence. The authorized Spanish version of the DUDIT was used only for participants who had used drugs in the last year.

Data collection and ethics

Study data were collected and managed using Research Electronic Data Capture (REDCap) at “Asociación Ideas for Health,” a Spanish non-profit organization focused on research and medical education [13]. The study was conducted in accordance with the Declaration of Helsinki, and the survey was voluntary and anonymous, eliminating the need for written informed consent. The Institutional Review Board and the Research Ethics Committee of Hospital General Universitario Gregorio Marañón approved the study (HUIL 1606-13/2019).

Statistical analysis

All analyses were performed using IBM SPSS v.24 (IBM Corp., Armonk, NY, USA). Two-tailed *p*-values were reported, with a significance level of *p* < 0.05.

The sample was described using absolute and relative frequencies for categorical variables and mean and standard deviation (SD) or median and interquartile range (IQR) for continuous variables. The chi-square test and independent samples *t*-test were employed in the univariate analysis to compare baseline characteristics between participants engaged and non-engaged in SDU. Multiple logistic regression analyses were conducted, with SDU as the dependent variable, to explore the association between SDU and variables related to baseline characteristics, high-risk sexual behaviors, and STIs. Independent variables were included if they showed a significant association with the dependent variable in the univariate analysis (*p* < 0.05). Age was included as a continuous variable in the regression analysis due to its potential influence on patterns of drug consumption. To assess the patterns and the changes in the prevalence of SDU and evaluate differences between the U-SEX-1 study (2016/2017) and the U-SEX-2 study (2019/2020), a comparative analysis was conducted using the study year as the independent variable.

## 3. Results

To detect potential nonresponse bias, we collected sociodemographic and clinical variables of the people who did not respond to the survey. There were no major differences between those who responded and those who did not in terms of age, foreign origin, had completed university studies, or illicit drug consumption (Table 1).

### 3.1. Participant Characteristics

A total of 424 HIV+ GBMSM were included in the present analysis. The mean age was 40 years (SD = 10.43). Participants were predominantly Spanish-born (*n* = 259, 61%), with 33% (*n* = 140) born in South and Central America. Over half (*n* = 232, 55%) had completed university studies. Overall, 63% (*n* = 266) had monthly incomes exceeding 1000 euros, and 73% (*n* = 304) were employed, whereas 13% (*n* = 54) were unemployed. The mean time since HIV diagnosis was 8.9 years (SD = 7.03), with 419 (99%) participants on antiretroviral therapy (ART). Of these, 96% reported an adherence rate of >90% to ART.

Approximately half of the sample (*n* = 194, 46%) reported having a stable partner, with 33% of these partners being HIV-positive. Overall, 396 (93%) reported being sexually active. Of these, 63% (*n* = 249) reported unprotected sex in casual partner relationships, with 21% (*n* = 84) never using condoms. Regarding high-risk sexual behaviors, in the previous year, 187 (44%) had had more than 24 sexual partners, 68 (16%) had practiced fisting, and 21 (5%) had engaged in transactional sex. Furthermore, 30% (*n* = 119) reported having had threesome sex, and 21% (*n* = 83) group sex (with 17% involving >6 individuals) (Table 2).

Overall, 313 (74%) participants had been diagnosed with an STI at any time, and 140 (33%) had received such a diagnosis in the preceding year. Of these recent diagnoses, 64% were syphilis, 26% chlamydia, 31% gonorrhea, 3% hepatitis A, and 5% hepatitis C. Most participants (n = 190, 61%) disclosed their diagnosis to their sexual partners and underwent contact tracing. In total, 229 (54%) participants reported using drugs in the previous year.

### 3.2. Sexualized Drug Use (SDU): Drug Consumption and Psychological Distress Symptoms

By the criteria defined in the Methods section, 25% of participants (*n* = 107) had engaged in SDU in the previous year. The most frequent drugs used during sex were mephedrone (*n* = 71, 66%), GHB (*n* = 71, 66%), cocaine (*n* = 44, 41%), crystal methamphetamine (*n* = 36, 34%), ketamine (*n* = 29, 27%), and MDMA (*n* = 27, 25%). Notably, 55.1% (*n* = 59) reported combining different drugs within a sexual context, with 27 (61%) of these regularly combining 3 or more drugs per session (polydrug use). A total of 17 participants (16%) reported slamsex, and 23 (21%) used drugs rectally, with mephedrone and other cathinones being the primary drugs used in rectal administration (*n* = 18, 78%). In addition to these substances, participants engaged in SDU reported having used erectile dysfunction drugs (*n* = 82, 76.6%), nitrites (*n* = 93, 90%%), sedatives, relaxants, or hypnotics (*n* = 44, 41.1%), protein supplements (*n* = 39, 36.4%), and anabolic steroids (*n* = 9, 8.4%) in the past year.

Regarding the location of SDU sessions, 98.1% reported practicing SDU in private homes, 43% in saunas or sex bars, 20.6% in nightclubs, and 7.5% in cruising areas. SDU sessions lasted over 12 h in 24.2% of the cases. More than 90% of the SDU-practicing participants used apps or websites to find sexual partners. Of these, 53.2% used social networks exclusively to find sexual partners for SDU.

Out of the 107 participants engaged in SDU, 101 participants completed the DUDIT-test, 81 (80%) scored ≥ 6 points, suggesting probable drug-related problems, with 8 (8%) indicating probable drug dependence (DUDIT score > 25). In terms of mental health, 18 participants engaged in SDU (17%) reported a diagnosis of depression, with 61% of these receiving pharmacological treatment. Moreover, 21 (20%) reported having been diagnosed with an anxiety disorder, 56% of whom were currently receiving pharmacological treatment. Three participants (2.8%) reported a diagnosis of a psychotic disorder, and another three (2.8%) a personality disorder. Finally, scores in the HADS questionnaire suggest that 37.4% (*n* = 40) had significant anxiety symptoms and 17.8% (*n* = 19) significant depressive symptoms.

### 3.3. Factors Associated with SDU

Compared to participants who were not engaged in SDU, those engaged were more likely Spanish-born, earn salaries greater than 1000 €/month, had incomplete adherence to ART, and were active smokers. Participants engaged in SDU also had higher proportions of risk behaviors and higher rates of STI (Table 2 and Table 3).

In the multivariate analysis, factors independently associated with SDU were having been diagnosed with an STI in the last year, aOR 3 (1.7–5.4), *p* < 0.001; having practiced fisting, aOR 6.1 (3.0–12.1), *p* < 0.001; unprotected anal intercourse, aOR 4.8 (1.9–12), *p* = 0.001; and having more than 24 sexual partners in the last year, aOR 2.1 (1.2–3.8), *p* = 0.008.

### 3.4. SDU in Two Time Periods

We compared the U-SEX-1 Study (2016/2017) and the U-SEX-2 Study conducted in 2019–2020. There were no notable differences observed between the two studies in terms of the prevalence of SDU (29% vs. 25%, *p* = 0.155) and slamsex (4.6% vs. 4.5%, *p* = 0.936). However, there were certain differences in the second survey compared to the first. The U-SEX-2 study reported a higher percentage of foreign participants (26% vs. 38%), an increased proportion of participants on ART (96% vs. 99%), and a greater number of individuals engaged in risky sexual behavior and STIs (Table 4). As for substance use, no significant differences were found between the two studies, except for cocaine that showed a decline between 2016 and 2020 (46% vs. 36%).

## 4. Discussion

The profile of the GBMSM with HIV who completed the questionnaires in our study were young, well educated, and had a high earning capacity. In total, 107 (25%) of the participants had engaged in SDU in the previous year and of these, 17 (16%) used drugs intravenously in the last year. SDU was associated with risky sexual behaviors and STIs. Among the participants who reported SDU in the past year, according to the DUDIT scales, 80% showed a risk of problematic drug use and 8% showed data indicative of drug dependence. Lastly, it was observed that the prevalence of SDU remains high among GBMSM with HIV, and the magnitude of this phenomenon has not significantly changed between the two measured time points (2016 and 2020) in Madrid.

The association between SDU and high-risk sexual behaviors has been recognized since the phenomenon’s initial identification [3,4,5,14,15,16,17,18]. This factor, alongside a reduced perception of risk, contributes to a direct correlation between SDU and STIs as has been consistently documented in the literature [3,4,14,15,16,19]. Consequently, SDU is associated with an increased incidence of STIs, including HIV and hepatitis C.

Our research identified a high prevalence of SDU and STIs in persons living with HIV, along with significant symptoms of depression, anxiety, and drug-related problems. These findings underscore the complexity and intersectionality of symptoms and behaviors in this population, necessitating a comprehensive, multidisciplinary approach to treatment [7,9,20,21].

In the present study, we replicated the cross-sectional U-SEX-1 Study [6], conducted in 2016–2017, to examine changes in prevalence and patterns of drug use or sexual practices among GBMSM living with HIV and engaged in SDU in Madrid, Spain. Participant characteristics in this study closely resembled those described in the U-SEX-1 study. As in the U-SEX-1 study, U-SEX-2 participants engaged in SDU were more likely to partake in high-risk sexual behaviors and were more frequently diagnosed with an STI than those not engaging in SDU. The use of different drugs remained constant over the years, except for cocaine, which seems to have decreased over time. Moreover, the practice of SDU appeared to sustain after four years in GBMSM living with HIV in Madrid (29% vs. 25%).

The stable prevalence rates at the two time points in Madrid could be interpreted as a sign that existing strategies may be maintaining the status quo, as we have not observed a significant increase in cases. However, this lack of change is concerning, given that the prevalence of SDU remains alarmingly high. Our data underscore the urgent need for new preventive actions aimed at reducing these rates. From a public health perspective, the rise in high-risk sexual behaviors in this population should serve as an alarm bell for healthcare institutions and policymakers. Creating working groups to establish and agree on the most appropriate strategies for prevention, risk reduction, and harm reduction in the population practicing SDU should be a priority. Increasing awareness among individuals practicing SDU through preventive measures is a task for public health and all professionals caring for GBMSM living with HIV.

In the context of SDU, there is a substantial body of research focused on prevalence rates and factors associated with this behavior in different parts of the world [4,6,15,16]. However, longitudinal data are crucial to understand the dynamics of this phenomenon, including shifts in consumption patterns and practices. Such insights are essential for tailoring and optimizing intervention strategies. Although limited, recent publications of longitudinal studies have shed light on the temporal evolution of SDU, revealing discernible trends and changes over time. For instance, a longitudinal study conducted in London and Brighton (AURAH2 study) examined changes in SDU and associated drug use over a three-year period (2015–2018) [22]. Despite indicating a decline in SDU prevalence (from 32% to 11.1%) and the use of chemsex-specific drugs (mephedrone declined from 25.2% to 9.7% and GHB/GBL use had also declined from 19.9% to 8.3%), these results diverge from our study’s findings, as we have found a slight but not significant decrease in the prevalence of SDU.

According to the Drug Use Disorders Identification Test (DUDIT) scales, 8 out of 10 participants exhibited a risk of problematic substance use, and nearly 8% displayed probable dependence. Although a clinical evaluation using DSM-5 or ICD-10 criteria is required for a definitive diagnosis, these questionnaires are sensitive, validated tools that provide an approximation of the risk of problematic use. Another frequent and concerning phenomenon is intravenous drug use (slamming), which in our study accounted for 16%. This figure is similar to the prevalence observed in other geographical regions [4,23,24,25]. Substance use disorders or problematic substance consumption also significantly interfere with various spheres of an individual’s life, including personal, family, and work relationships. Within the context of this population, health consequences such as STIs and other infections should also be considered as indicators of problematic use.

In terms of management, early detection of problematic use is crucial. There is a window in which problematic use can be suspected or identified before it progresses to a substance use disorder. Proper management of this syndemic, along with promoting self-care, could enhance these individuals’ perception of health and their overall quality of life [26,27].

The utility of questionnaires in assisting social health personnel in detecting potential problematic use is still to be determined [12]. Once suspected, it should be confirmed and referred to specialized care. However, the sociosanitary resources available for treating individuals with problematic SDU are often limited.

It is also vital to investigate the factors that initiate and perpetuate these problematic practices to identify vulnerable GBMSM living with HIV and to implement preventive strategies. Our research found a relatively high prevalence of SDU and STIs in persons with HIV in the population, accompanied by significant symptoms of depression, anxiety, and drug-related problems. These findings highlight the complex and intersecting symptoms and behaviors in this population, necessitating a holistic, interdisciplinary approach to treatment [7,9,20,21].

Thus, HIV units and sexual health clinics should provide comprehensive assessment and care, including STI and drug use prevention, being directly coordinated with the specific drug and mental health services. In our view, traditionally effective measures to prevent STIs, drug use, and mental health problems among GBMSM who use drugs should be combined to develop interdisciplinary programs. However, new prevention strategies are needed to address the needs of specific populations at higher risk of STIs and to demonstrate effective interventions in specific populations at higher risk of STIs, drug-related, and mental health problems [28]. Furthermore, all these services should continue to adapt, with their professionals receiving specific training to successfully attend to this population [8].

In Madrid, initiatives have been launched by the public health service of the Health Council. An advisory group has been formed consisting of NGOs, healthcare providers, and personnel from STI or HIV clinics and addiction centers. The goal is to implement a series of measures to educate the target population and work in an interdisciplinary manner.

Our study has certain limitations inherent to cross-sectional survey studies, especially response bias and these types of studies cannot explain causality. Although we used limited time periods in questions that depended on memory, recall bias could distort the accuracy of the results. Nonetheless, descriptive/analytical cross-sectional studies are useful for establishing preliminary evidence for a causal relationship, and furthermore, longitudinal monitoring could help identify trends. Unfortunately, the study had to be halted prematurely in February 2020 due to the COVID-19 pandemic, a decision made by the scientific coordinators considering the likely impact of the pandemic on the study’s objectives. This led to our inability to recruit all the initially planned participants. However, the final number of participants is considered sufficient for conducting the study. Our study has several strengths. Firstly, we report, for the first time, data on the prevalence of SDU over time in GBMSM living with HIV in Spain. This could allow our findings to be used to infer about the potential effectiveness of the interventions that have been developed for this population in our country. Our research also benefits from a well-defined and large sample size. This was made possible by the eligibility criteria being applied by the patients’ own physicians, who were able to collect baseline information and distribute the survey directly.

## 5. Conclusions

Our study revealed that sexualized drug use (SDU) continues to be prevalent in Spain among GBMSM living with HIV. Additionally, through the application of the Drug Use Disorders Identification Test (DUDIT), we have identified a high prevalence of potential problematic substance use and possible substance dependence within this demographic. The pattern and prevalence of SDU have remained relatively constant over time. These results highlight the critical need for ongoing, focused interventions that tackle SDU, sexual health, mental health, and substance use issues among this group.

## Figures and Tables

**Table 1:** Differences between patients who responded to the questionnaire and those who did not.

	Did Not Respond to the Questionnaire (588)	Responded to the Questionnaire (412)	*p*-Value
Age, median (IQR)	39 (33–47)	39 (33–47)	0.655
Foreigner, *n* (%)	217 (37.0%)	153 (37.4%)	0.903
University studies, *n* (%)	287 (49.4%)	226 (55.4%)	0.063
Had ever used drugs, *n* (%)	382 (69%)	299 (72%)	0.052

Abbreviation: IQR: interquartile range.

**Table 2:** Characteristics of the patients included in the analysis stratified by sexualized drug use.

	Total (*n* = 424)	No SDU (*n* = 317)	SDU (*n* = 107)	*p*-Value
Age, *M* (*SD*)	40 (10.43)	40.53 (11.10)	38.42 (7.99)	0.034
Spanish-born, *n* (%)	259 (61.5%)	183 (57.7%)	76 (71%)	0.013
University studies, *n* (%)	232 (55.2%)	169 (53.3%)	63 (58.8%)	0.315
Salary > 1000 euros/m, *n* (%)	266 (63.3%)	189 (59.6%)	77 (71.9%)	0.021
Years since HIV diagnosis, *M* (*SD*)	8.88 (7.03)	9.22 (7.39)	7.89 (5.80)	0.059
On ART, *n* (%)	419 (99.3%)	312 (98.4%)	107 (100%)	0.311
Complete adherence (>95%) to ART, n (%)	394 (93%)	299 (94.3%)	100 (94%)	0.431
Stable partner, *n* (%)	194 (45.7%)	155 (48.8%)	39 (36.4%)	0.030
Depression, *n* (%)	64 (15%)	46 (14.5%)	18 (16.8%)	0.564
Anxiety, *n* (%)	63 (14.8%)	42 (13.2%)	21 (19.6%)	0.109
Substance abuse, *n* (%)	26 (6.1%)	8 (2.5%)	18 (16.8%)	<0.001
Self-reported HIV-related distress, *M* (*SD*)	33.51 (31.08)	36.14 (31.60)	25.74 (28.23)	0.005
Anxiety symptomatology, *M* (*SD*)	6.00 (4.07)	5.91 (4.05)	6.25 (4.12)	0.466
Depression symptomatology, *M* (*SD*)	3.56 (3.70)	23.57(3.62)	3.55 (3.94)	0.972
Risk behaviors				
Sexually active in the last year, *n* (%)	396 (93.6%)	289 (91.5%)	107 (100%)	0.002
≥24 sexual partners, *n* (%)	187 (47.2%)	114 (39.4%)	73 (68.2%)	<0.001
Unprotected anal intercourse, *n* (%) ¥	272 (69%)	172 (60%)	100 (93%)	<0.001
Transactional sex, *n* (%)	21 (5%)	13 (4%)	8 (7%)	0.250
Fisting, *n* (%)	68 (17.3%)	21 (7.3%)	47 (44.3%)	<0.001
Sexually transmitted infections				
Any STI, *n* (%) ɛ	313 (73.8%)	212 (66.9%)	101 (94.4%)	<0.001
Any STI in the previous year, *n* (%) ɛ	140 (33%)	72 (22.7%)	68 (63.6%)	<0.001
Syphilis, *n* (%) ɛ	89 (21%)	40 (12.6%)	49 (45.8%)	<0.001
Gonorrhea, *n* (%) ɛ	44 (10.4%)	23 (7.3%)	21 (19.6%)	<0.001
Chlamydia, *n* (%) ɛ	37 (8.7%)	18 (5.7%)	19 (17.8%)	<0.001
Hepatitis C, *n* (%) ɛ	7 (1.7%)	1 (0.3%)	6 (5.6%)	<0.001
Urethritis, *n* (%) ɛ	24 (5.7%)	9 (2.8%)	15 (14%)	<0.001
Proctitis, *n* (%) ɛ	17 (4%)	10 (3.2%)	7 (6.5%)	0.122

Notes: ¥, with casual partners; ɛ, self-referred diagnosis. Statistical analysis: differences were evaluated using the chi-square test or Fisher’s exact test for categorical variables and with a *t*-test for independent samples for quantitative variables. Abbreviations: *M*, mean; SDU, sexualized drugs use; ART, antiretroviral therapy; HIV, human immunodeficiency virus; *SD*, standard deviation; STI, sexually transmitted infections.

**Table 3:** Characterization of drug use in the overall population and in the population stratified by sexualized drug use.

Drug Use in the Last Year	Total (*n* = 424)	No SDU (*n* = 317)	SDU (*n* = 107)	*p*-Value
Active smoker, *n* (%)	148 (35.2%)	92 (29.3%)	56 (52.8%)	0.001
Regular drinker (≥3 times/week), *n* (%)	55 (13%)	36 (11.4%)	19 (17.8%)	0.088
Ever used drugs, *n* (%)	306 (72.2%)	199 (62.8%)	107 (100%)	<0.001
Used drugs in the last year, *n* (%)	241 (56.8%)	134 (42%)	107 (100%)	<0.001
Cannabis, *n* (%)	130 (46.4%)	70 (39.3%)	60 (58.8%)	0.002
Nitrites, *n* (%)	183 (65.6%)	90 (51.1%)	93 (90.3%)	<0.001
Cocaine, *n* (%)	101 (36.2%)	31 (17%)	70 (72.2%)	<0.001
GHB/GLH, *n* (%)	89 (33.7%)	11 (6.8%)	78 (75.7%)	<0.001
Mephedrone, *n* (%)	85 (32.3%)	9 (5.6%)	76 (74.5%)	<0.001
MDMA, *n* (%)	83 (31.3%)	29 (17.1%)	54 (56.8%)	<0.001
Crystal meth, *n* (%)	45 (17.9%)	5 (3.1%)	40 (43.5%)	<0.001
Ketamine, *n* (%)	53 (20.5%)	13 (8%)	40 (42.1%)	<0.001
Slamsex, *n* (%)	19 (4.5%)	2 (0.6%)	17 (15.6%)	<0.001
Combination ≥ 3 drugs, *n* (%)	27 (6.4%)	0 (0%)	27 (25.2%)	<0.001
Used apps for sex and drugs, *n* (%)	214 (70.6%)	116 (59.2%)	98 (91.6%)	<0.001
Intrarectal drug use, *n* (%)	23 (5%)	0 (0%)	23 (21%)	<0.001
Risky drug use (DUDIT ≥ 6 points), *n* (%)	122 (54.7%)	41 (33.6%)	81 (80.2%)	<0.001
Drug dependence (DUDIT ≥ 25 points), *n* (%)	8 (3.6%)	3 (2.5%)	5 (5%)	0.473
Sedatives/tranquilizers, *n* (%)	109 (25.8%)	65 (20.6%)	44 (41.4%)	<0.001
Erectile dysfunction drugs, *n* (%)	145 (34.4%)	63 (20%)	82 (76.6%)	<0.001

Statistical analysis: differences were evaluated using the chi-square test or Fisher’s exact test for categorical variables. Abbreviations: SDU, sexualized drugs use; GHB/GLH, γ-hydroxybutyrate/γ-butyrolactone; MDMA, 3,4-methylenedioxy-N-methylamphetamine; crystal meth, crystal methamphetamine; slamsex, intravenous SDU; DUDIT, Drug Use Disorders Identification Test.

**Table 4:** Comparison of the U-SEX-1 study (2016/2017) and the U-SEX-2 study (2019–2020).

Characteristics	2016/2017 Usex-1	2019/2020 Usex-2	*p*-Value
Age, median (IQR)	38 (32–45)	40 (33–47)	0.328
Spanish-born, *n* (%)	545 (73.9%)	259 (61.5%)	<0.001
University-level studies, *n* (%)	438 (59.5%)	232 (55.2%)	0.157
Salary > 1000 euros/month, *n* (%)	478 (65.7%)	266 (63.3%)	0.409
Years from HIV diagnosis, median (IQR)	5 (2–10)	7 (3–12)	<0.001
On ART, *n* (%)	677 (95.8%)	419 (99.3%)	0.001
Complete adherence to ART, *n* (%)	524 (78.3%)	306 (73.4%)	0.078
Stable partner, *n* (%)	363 (49.3%)	194 (45.9%)	0.266
Risk behaviors			
≥24 sexual partners, *n* (%)	126 (18.6%)	187 (44.1%)	<0.001
Unprotected anal intercourse, *n* (%)	415 (55.9%)	272 (69.2%)	<0.001
Any STI, *n* (%)	465 (62.7%)	313 (73.8%)	<0.001
Fisting, *n* (%)	125 (16.8%)	68 (17.3%)	0.860
Sexual worker	21 (3.9%)	21 (5.3%)	0.304
Used apps for sex and drugs	307 (83.4%)	214 (70.6%)	<0.001
Use of drugs			
Ever used drugs, *n* (%)	518 (69.8%)	306 (72.2%)	0.395
Combination ≥ 3 drugs, *n* (%)	98 (13.2%)	27 (6.4%)	<0.001
Chemsex, *n* (%)	216 (29.1%)	107 (25.2%)	0.155
Slamsex, *n* (%)	34 (4.6%)	19 (4.5%)	0.936
Drugs used in the last year			
Cannabis	220 (46.0%)	130 (46.4%)	0.914
Nitrites	314 (63.8%)	183 (65.6%)	0.622
GHB	168 (36.7%)	89 (33.7%)	0.422
Cocaine	225 (46.4%)	101 (36.2%)	0.006
Mephedrone	162 (34.8%)	85 (32.3%)	0.503
MDMA	138 (30.4%)	83 (31.3%)	0.796
Cristal meth	67 (15.1%)	45 (17.9%)	0.330
Ketamine	89 (20.1%)	53 (20.5%)	0.886

Statistical analysis: differences were evaluated using the chi-square test or Fisher’s exact test for categorical variables and with a Kruskal–Wallis *U*-test for quantitative variables. Abbreviations: HIV, human immunodeficiency virus; ART, antiretroviral therapy; STI, sexually transmitted infections; GHB, ϒ hydroxybutyrate; MDMA, 3,4-methylenedioxy-N-methylamphetamine; crystal meth, crystal methamphetamine. Information regarding adherence to antiretroviral therapy (ART) was self-reported by participants. It was recorded as the number of days the medication was not taken in the last month. Incomplete adherence refers to having more than three missed doses per month.

## Data Availability

The data presented in this study are available on request from the corresponding author. The data are not publicly available due to privacy concerns.
